# Messages and Notifications for the “OA Coach” Knee Osteoarthritis Self-Management Mobile App: Codevelopment and Evaluation Using a Participatory Research Design With Focus Groups and Surveys

**DOI:** 10.2196/83507

**Published:** 2026-05-04

**Authors:** Kate L Bryce, Jocelyn L Bowden, David J Hunter, Robin Huang, Naomi Bloul, Vicky Duong, Jillian Eyles

**Affiliations:** 1 Sydney Musculoskeletal Research Centre, Kolling Institute, The University of Sydney, Level 10, Reserve Road, St Leonards, Sydney, NSW, 2065, Australia, 61 402446268; 2 Department of Rheumatology, Royal North Shore Hospital, Northern Sydney Local Health District, Sydney, NSW, Australia; 3 Vulsen, Sydney, NSW, Australia

**Keywords:** knee osteoarthritis, mobile app, digital health, notifications, education, self-management, behavior change, co-design, mixed methods

## Abstract

**Background:**

The OA Coach mobile app is a digital self-management tool designed to support people with knee osteoarthritis (OA).

**Objective:**

We aimed to enhance the digital user experience of the OA Coach app by working with consumers, health professionals, and researchers to (1) improve existing OA Coach app call-to-action and encouragement notifications by applying behavior change theory, refining, reviewing, and adapting them, and (2) co-design new educational messages based on international OA guidelines.

**Methods:**

This mixed methods study used a 3-phase process for improving existing notifications and developing new educational messages: (1) initial development and/or enhancement using behavior change techniques and accessible language, (2) expert review, and (3) refinement based on participant feedback. We enhanced the existing notifications to incorporate behavior change theory and improve readability, then we assessed ease of understanding, usefulness, and motivational impact in an online survey using 1‐5 Likert scales and free-text responses. Median scores and IQRs were calculated, free-text responses were summarized using content analysis, and notifications that scored ≤12/15 and/or had 2 or more consistent recommendations in free-text responses were systematically refined. The new weekly educational messages were initially drafted based on OA clinical practice guidelines, then reviewed in 3 focus groups (in person and online) for detailed guidance on topic selection, language, and timing. Content analysis of focus group data informed systematic refinement of the messages. The refined messages were assessed for understanding and usefulness in an online survey (1‐5 Likert scales), and free-text responses were refined further based on survey results.

**Results:**

Fifty-seven participants assessed the existing notifications in the survey. Consumers rated notifications relating to sleep and mood the lowest, and those encouraging logging pain scores the highest. Health professionals and researchers reported a median score of 12/15 across all notifications, with greatest variability observed for step count, activity tracker use, and pain score logging. Eighty notifications were refined to enhance clarity, engagement, and effectiveness in prompting meaningful actions by future OA Coach users. Overall, 6 consumers, 7 health professionals, and 6 researchers participated in 3 focus groups to co-design 14 educational messages. A preference for a consistent message structure emerged: a descriptive heading, a brief introductory sentence, followed by 3 or 4 key points with practical examples, and concluded with a motivating statement. Key themes guiding modifications for both notifications and educational messages included reducing technical jargon, simplifying colloquial expressions, and removing language perceived to be patronizing or frustrating.

**Conclusions:**

Through an iterative process, we used behavior change theory and worked with consumers, health professionals, and researchers to identify key preferences for message content, language, tone, and structure. These insights informed the refinement of 80 notifications and the co-design of 14 educational messages for the OA Coach app.

## Introduction

An estimated 595 million people globally have osteoarthritis (OA), most commonly at the knee and hip [1], with prevalence projected to surge dramatically by 74.9% and 78.6%, respectively [2]. Driven by an aging population, a rise in obesity, joint injuries, and increasing physical inactivity, OA is the fastest-growing cause of disability worldwide [1,3,4]. OA symptoms include joint pain, physical limitations, and associated challenges such as low mood, interrupted work, and social participation, resulting in enormous social and economic burden [5]. To address the enormous burden of OA, international clinical practice guidelines recommend education for self-management, exercise, and weight management as first-line interventions for all people with hip and knee OA [6-14]. This care can result in some people delaying or avoiding joint replacement surgery altogether [11,15-17]. However, in Australia, first-line OA treatments are underused [18,19], often driven by common misconceptions about OA, the perceived efficacy (or lack thereof) of first-line treatments, and limited access to these treatments [20,21].

Scalable solutions are needed to address these challenges that improve public access to guideline-recommended care. Digital health technologies, such as mobile health (mHealth) apps, offer a promising approach to overcome these barriers by providing accessible, low-cost, and evidence-based interventions that can be delivered at scale. Digital technology using mobile phones is a fast-growing field that promotes adherence to treatment in self-managing chronic conditions, including OA [22-25]. The World Health Organization identified developing digital health as a global priority, emphasizing that it can support equitable and universal access to quality health services, improve health outcomes [4,26], and improve access to the best-evidence care.

The OA Coach is a mobile app developed by the University of Sydney’s Osteoarthritis Clinical Research Group (OACRG), which aims to deliver first-line OA treatments and facilitate long-term behavior change for self-management in people with knee OA [27-30]. The OA Coach combines several strategies to achieve this, including the delivery of call-to-action and encouragement notifications via short, automated messages designed to prompt participants to take specific actions, such as logging their pain levels or completing a task. The knee OA education messages, on the other hand, are more detailed communications that provide participants with information, guidance, and support related to managing their condition. An initial bank of 88 notifications were developed by the researchers (VD and NB) and were piloted in a feasibility study (March-April 2024) [27]. Although the messages used positive, encouraging language, they were not grounded in behavior change theory. Participant feedback from the pilot study suggested that the notifications were useful, though improvements in content would enhance their effectiveness.

Therefore, we aimed to enhance the digital user experience of the OA Coach app by improving the relevance and utility of the notifications by incorporating behavior change theory, data-driven techniques, and a co-design approach. Longer educational messages were also developed to better support participants to self-manage their knee OA.

The two aims were (1) to enhance the original OA Coach app call-to-action and encouragement notifications by applying behavior change theory, refining, reviewing, and adapting them with consumers, health professionals, and researchers, and (2) to codevelop new knee OA education messages based on international OA guidelines using focus groups and surveys with consumers, health professionals, and researchers.

## Methods

### Study Design

The mixed methods study design was based on 2 previously published frameworks for digital intervention development [31,32] and adapted to include three phases: (1) initial development and/or enhancement with behavior change techniques (BCTs) and accessible language, (2) expert review, and (3) refinement based on participant feedback. The study was conducted at the Kolling Institute, the University of Sydney (December 2024 to March 2025). This study is reported in accordance with STROBE (Strengthening the Reporting of Observational Studies in Epidemiology) guidelines (Checklist 1) [33].

### The OA Coach App

OA Coach integrates a wrist-based activity tracker (Fitbit Inspire 2) to monitor physical activity levels, calorie count, and hours of sleep. The app incorporates recommended first-line OA management, including education for self-management and strategies for increasing physical activity levels. It uses BCTs, defined as the smallest components within intervention strategies that are observable and replicable, and designed to facilitate a shift in health behaviors [34]. Examples of BCTs used in the OA Coach app are self-monitoring of pain levels and body weight, call-to-action and encouragement notifications, and goal setting to improve and sustain physical activity levels.

The OA Coach app integrates both passive data collection (eg, step count, sleep duration, and energy expenditure/calories burned via the Fitbit Inspire 2) and active user-reported measures (eg, pain scores on a visual analog 1‐10 scale [35], 21-Item Depression, Anxiety and Stress Scale (DASS-21) [36,37], Pittsburgh Sleep Quality Index (PSQI) [38], and body weight tracking). This combination enables the delivery of tailored feedback and support consistent with the principles of digital behavior change interventions, which use technology to promote health behavior change through personalized and timely strategies [39,40]. In particular, the app uses just-in-time adaptive interventions (JITAIs) design principles [41], in which prompts and guidance are delivered at moments when they are expected to be most relevant and actionable, based on predefined rules using the user’s current context, progress, and goals [41]. In the OA Coach app, these JITAI-informed interventions are rule-based. For example, if the user takes fewer than their goal number of steps by 4 PM, then at 5 past the hour, a call-to-action notification is sent: “Almost there! [number of steps] steps stand between you and the step podium. Strap on those sneakers and step into the winner’s circle.”

### Participants and Recruitment

People were eligible if they had been diagnosed with knee OA, were aged 18 years or older, spoke fluent English, and owned a smartphone. Previous participants from the feasibility study were contacted to be part of this study. Consumers may or may not have used the OA Coach app previously during the 6-week feasibility study [27]. Health professionals and researchers were eligible if they had experience working with people with knee OA. Specifically, health professionals were eligible if they treated people with OA, and researchers were expected to have published at least two research papers on OA management. Demographic data collected included age, gender, ancestry, education level, and years of work experience (health professionals and researchers).

Recruitment was conducted through the OACRG volunteer database. Health professionals and researchers were invited via email through existing professional networks. Purposeful sampling for the focus groups was used to ensure diversity in participant ethnicity, age, professional role, and educational level. Participation in the focus group was not dependent on completing the survey.

### Aim 1: The Enhancement of Call-to-Action and Encouragement Notifications With Consumer, Health Professional, and Researcher Review

#### Phase 1: Enhancement of Notifications With BCTs and Accessible Language

First, using the pilot data and existing evidence for common barriers and facilitators to exercise program uptake in individuals with knee OA [42-45], we reviewed the content and purpose of each existing OA Coach app notification to identify its core message and assign a behavioral outcome. The notifications were divided into 6 domains: step count, sleep goals, logging pain scores, completing sleep and mood surveys, goal achievement, and wearing an activity tracker. They were also categorized as one of two types: “call-to-action” notifications designed to prompt a specific behavior, or “encouragement” notifications, intended to motivate the user.

Second, the notifications were individually mapped to the Behavior Change Technique Taxonomy (v1) [46]. Some notifications were modified to incorporate additional, appropriate BCTs such as information about health consequences (BCT 5.1), instructions on how to perform a behavior (4.1), goal setting (1.1), action planning (1.4), and habit formation (8.3) [46]. A summary of the specific BCTs applied across the notifications is provided in Multimedia Appendix 1.

Third, the notifications were modified to target a reading level of grade 8 or lower to reduce reading effort and to help prevent potential misunderstandings for all users (approximately 12‐14 years of age) [47]. The Sydney Health Literacy Lab’s SHeLL Editor was used to support simplification without compromising meaning [48,49]. Table 1 shows examples of the initial notification enhancement process, with the literacy level score based on the SHeLL Editor [49].

**Table 1.** Example of the initial notification enhancement process. Drafts were revised by integrating behavior change techniques (coded using the Behavior Change Technique Taxonomy v1) and assessed for readability (Sydney Health Literacy Editor; target ≤ grade 8).

Initial notification	Summary of content	Additional content	BCTTv1^a^ classification	Reading level	Enhanced notification
You’re on fire! Keep those steps coming. 🔥 🚶	General encouragement and support	Add detailed instructions on how to succeed	Prompt/cues (BCT^b^ 7.1), social support (unspecified) (BCT 3.1), habit formation (BCT 8.3), action planning (BCT 1.4)	3.1	You’re on fire! Keep those steps coming. 🔥🚶Make it a habit by walking at the same time each day
🚶 Look at you go! You exceeded your step goal.	Immediate feedback using positive language informing user they have met their step goal	Add information about health consequences	Prompt/cues (BCT 7.1), feedback on behavior (BCT 2.2), social support (unspecified) (BCT 3.1), information about health consequences (BCT 5.1)	7.8	🚶 Look at you go! You exceeded your step goal. Your knees are getting stronger, and you’ve improved your overall joint health
📅 Time for a sleep check-up! Your PSQI^c^ survey is waiting. Let’s make sure your nights are as restful as they can be! 🌜 💤	Prompt and feedback on health consequences implying monitoring sleep will make nights more restful	Negative feel to message. PSQI to change to sleep survey to enhance clarity of notification. Add instruction on how to perform the behavior (filling in the survey)	Prompt/cues (BCT 7.1), information about health consequences (BCT 5.1), instruction on how to perform the behavior (BCT 4.1), social support unspecified (BCT 3.1)	8.8	📅 Ready to enhance your sleep quality? Fill in your sleep survey now to optimize your nights for restful sleep! 🌜💤 Your input makes a difference!
🎉 Great job wearing your Fitbit this week!	Prompt and encourages continued engagement (commitment to Fitbit wear) with positive reinforcement	Add information about health consequences	Prompt/cues (BCT 7.1), feedback on behavior (BCT 2.2), social support unspecified (BCT 3.1), information about health consequences (BCT 5.1)	7.2	🎉 Great job wearing your Fitbit this week! Every step counts toward your health goals!🚶 🚶

a BCTTv1: Behavior Change Technique Taxonomy v1.

b BCT: behavior change technique.

c PSQI: Pittsburgh Sleep Quality Index.

#### Phase 2: Consumer, Health Professional, and Researcher Review

We planned to recruit 40 consumers, 10 health professionals, and 10 researchers to allow for each of the 80 notifications to be reviewed by a minimum of 5 consumers and 2 health professionals or researchers, distributed across 8 discrete surveys. The 8 surveys were automatically randomly allocated to participants using the Research Electronic Data Capture (REDCap) system [50,51]. Each notification was assessed based on 3 questions using a 5-point Likert rating scale used in similar studies in cardiovascular disease and low back pain [31,32]. The questions were:

1. Is the information in this notification easy to understand? (“1=very difficult to understand” to “5=very easy to understand”)
2. Is the information in this notification useful? (“1=not useful at all” to “5=very useful”)
3a. How likely are you to take action after receiving this notification? (“1=not likely at all” to “5=very likely”)
3b. How would you rate the positive encouragement within this notification? (“1=very poor” to “5=excellent”)

There was also a free-text section for general feedback and suggestions for language modifications. Scores for each message were summed to yield a total score between 3 and 15 points (15 was best). This scoring system was a descriptive tool used to guide refinements of the notifications and to support interpretation, rather than an indicator of effectiveness or being intended as a validation tool.

#### Phase 3: Data Analysis and Refinement

Descriptive statistics (median and IQR) were calculated for 3 of the 4 characteristics (understanding, usefulness, likelihood of taking action, and positive encouragement) within each notification domain (see the Results section). Results were analyzed separately for consumers and for health professionals/researchers, then combined. All analyses were conducted in Microsoft Excel and manually cross-checked for accuracy. Visualizations, including radar charts (Multimedia Appendix 2), were created to highlight group-based patterns (consumer vs health professionals/researchers) and domain-specific performance. Qualitative feedback from open-ended survey responses was summarized using a simple content analysis approach. Comments were coded into common categories, including tone, clarity, grammatical accuracy, and perceived helpfulness.

Thresholds for perceived notification acceptability were established to assist with the interpretation of the survey data based on approaches used in prior research [31,32]. Median scores below 12 were classified as low acceptability, scores of 12 and 13 indicated moderate acceptability, and scores of 14 and above as high acceptability. Notifications that received a median score of <12 points were reviewed and revised based on feedback. Notifications that scored ≥12 points were modified if minor changes were suggested or if there was consistent feedback from 2 or more participants.

These insights guided the final refinement of notifications, even for those that met the quantitative threshold of 12/15 or more.

### Aim 2: The Development of Knee OA Educational Messages

#### Phase 1: Development and Enhancement of Educational Messages With BCTs and Accessible Language

We drafted 12 new educational messages, designed to be longer and more informative than the existing call-to-action and encouragement notifications, incorporating clinical practice guideline recommendations and 21 key patient messages about OA established by French et al [7,52,53]. The messages featured BCTs developed using the capability, opportunity, motivation—behavior (COM-B) model and the Behavior Change Technique Taxonomy [46,54,55]. The Flesch-Kincaid readability score [56] and SHeLL Editor [49] were used to evaluate the readability of the educational messages and identify opportunities to simplify the language and sentence structure to align with a grade 8 reading level. We planned to deliver one educational message weekly to the OA Coach users’ app inbox over a 12-week period.

#### Phase 2: Consumer, Health Professional, and Researcher Review

To explore the educational messages in-depth, we conducted 3 focus groups, aiming to recruit 6 participants per group as recommended to encourage rich, detailed feedback [57,58], and allow for collaborative rewriting of the message content. The first focus group for consumers was 3 hours in duration, held in person at the Kolling Institute, University of Sydney. The second and third focus groups were 2 hours in duration, held online with health professionals and researchers, respectively. Our focus groups were structured around the 7-step co-design framework established by Trischler et al [59], which our team used previously [60]. A semistructured discussion guide was drafted by the lead author (KLB) (Multimedia Appendix 3). The guide aimed to explore participant views on whether the messages aligned with international guidelines (health professionals and researchers only), incorporated positive language and BCTs, provided practical strategies for lifestyle changes, and the optimal order and timing of messages within the app. The guide was reviewed and refined by 2 coauthors (JE and JLB) prior to use. The focus group methodology was informed by the information power approach, whereby researchers prioritized depth and richness of discussion of each message rather than aiming for data saturation [61]. Three focus groups were sufficient for this approach.

Audio recordings and verbatim transcriptions of the focus group discussions provided detailed insights into group dialogue. For the in-person focus group with consumers, printed posters displaying each message were provided, and participants were encouraged to add Post-it notes with suggestions to improve them. Similarly, during the online focus groups with health professionals and researchers, a shared digital whiteboard was used to record participants’ comments on each message in real time. Focus group participants were unable to view others’ comments.

#### Phase 3: Data Analysis and Refinement

We conducted a content analysis of focus group data, including audio-recorded transcripts from the 3 focus groups and feedback written directly on printed message handouts, posters, and online Post-it notes within Microsoft Whiteboard. One researcher (KLB) manually reviewed and categorized all data in a Microsoft Excel spreadsheet, grouped participant comments by message, and noted recurring patterns and points of emphasis, particularly related to clarity, tone, and usefulness. The categorization of the messages was reviewed by a second researcher (JE). Using a structured approach, each message was systematically refined and checked by the research team.

Following this, the focus group participants were sent a final REDCap survey to evaluate the refined education messages. Participants were asked to rate how easy each message was to understand (“1=very difficult to understand” to “5=very easy to understand”) and its usefulness (“1=not useful at all” to “5=very useful”). Participants also had the opportunity to provide further feedback for each educational message. This feedback was used to refine the educational messages a final time before they were added to the bank of automated educational messages in both audio and readable formats within the OA Coach mobile app.

Given the small sample size for the final survey, we used median values to represent central tendency and reported the minimum and maximum scores to describe the full range of responses. Results were analyzed separately for consumers and for health professionals/researchers, then combined. All quantitative data were analyzed using Microsoft Excel.

### Ethical Considerations

Ethics approval was provided by the University of Sydney Human Research Ethics Committee (2024/HE001089). All participants provided informed consent. Participant data were deidentified, securely stored, and confidentiality was maintained throughout analysis and dissemination. Consumer participants received AUD 150 (US $106.92) and parking costs for in-person focus group participation as recommended by Health Consumers NSW [62]. Consumers who completed the notification survey were placed in a draw to win one of two AUD 100 (US $71.28) vouchers. The health professional and researcher participants were not remunerated.

## Results

### Participants

**Figure 1.**
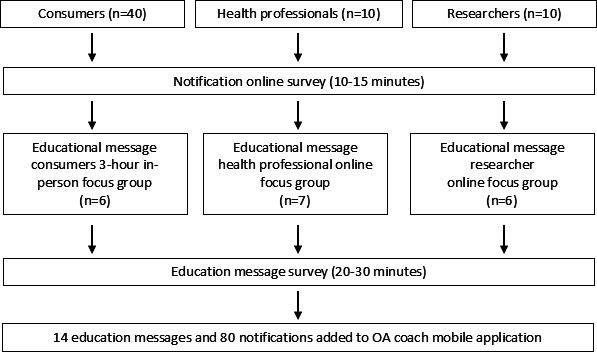
Flow of participants through notification and education message evaluation stages.

Figure 1 illustrates the flow of participants throughout the study, including engagement with the notification survey, focus groups, and a final educational message survey. A total of 40 consumers, 8 health professionals, and 9 researchers completed the initial online notification survey. Six consumers from the survey group also participated in the in-person consumer focus group. In addition, 7 health professionals and 6 researchers participated in their respective online focus groups. All focus group participants completed a final educational message survey following their group discussion.

Among consumers, 50% (20/40) identified as female, and the age range was 46 to 85 years. Educational attainment was high, with nearly half completing postgraduate study. The health professional and researcher group was predominantly female, with an age range of 42 to 56 years. This group was highly educated and experienced; the majority held postgraduate qualifications and had over 10 years of work experience. Further demographic data can be found in Table 2.

**Table 2.** Demographic details of participants included in the study.

	Consumers		Health professionals^a^ and researchers	
	Total (n=40)	Focus group (n=6)	Total (n=20)	Focus group (n=13)
Gender				
Woman, n (%)	20 (50)	3 (50)	15 (75)	11 (85)
Man, n (%)	20 (50)	3 (50)	5 (25)	2 (15)
Ancestry				
White (Australian, British Isles, European, and American), n (%)	38 (95)	5 (83)	17 (85)	11 (85)
Other (Middle Eastern and Asian), n (%)	2 (5)	1 (17)	3 (15)	2 (15)
Age (years)				
Mean (SD)	65 (8)	73 (8.6)	44 (7)	42 (6)
Range	46‐85	55‐79	42‐56	33‐55
Education, n (%)				
Three or more years of high school education	7 (18)	1 (17)	0 (0)	0 (0)
University degree	15 (38)	2 (33)	1 (5)	1 (8)
Postgraduate study	18 (45)	3 (50)	19 (95)	12 (92)
Years of work experience (health professionals and researchers only)				
3‐5 years	—^b^	—	2 (10)	1 (8)
6‐10 years	—	—	3 (15)	4 (31)
11‐15 years	—	—	5 (25)	3 (23)
Over 15 years	—	—	10 (50)	5 (38)

a Of the 10 health professionals, 9 were physiotherapists and 1 was a medical doctor.

b Not applicable.

### Aim 1: The Enhancement of Call-to-Action and Encouragement Notifications With Consumer and Expert Review

#### Consumer, Health Professional, and Researcher Review

A total of 80 notifications were assessed in the surveys. Of these, 16% (n=13) were scored below the 12/15 threshold and were revised based on participant feedback. In addition, 59% (n=47) of notifications with scores 12/15 or higher were refined in response to open-text feedback, which highlighted areas for improvement in clarity, tone, and grammatical accuracy. Nearly 20 (25%) notifications were retained unchanged, having scored 12/15 or higher, and received positive feedback.

**Figure 2.**
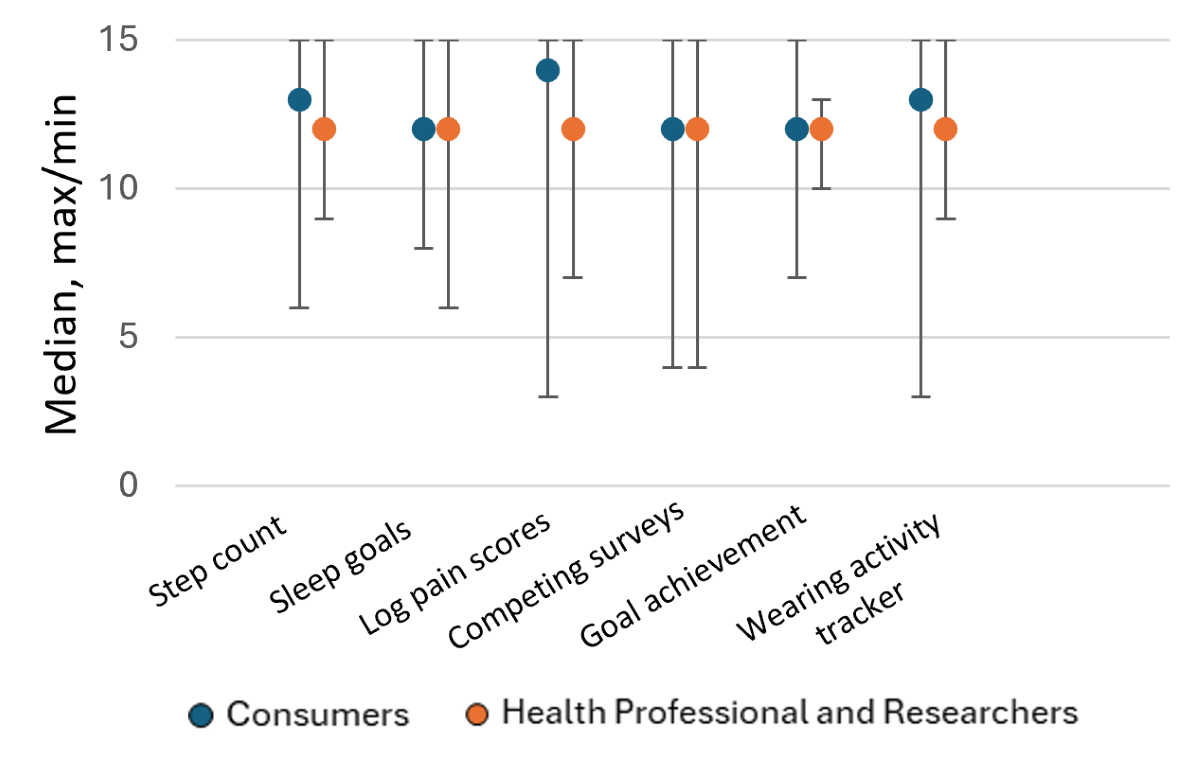
Total notification scores (out of 15) for the consumers and the health professional and researcher group. Distribution of total median scores for each notification domain with comparisons between the consumer group and the health professional and researcher group. Median scores are presented alongside minimum and maximum values. The scores range from 0 to 15. Higher scores indicate more positive evaluations.

Overall, participants provided generally high ratings across all notification domains, with subtle differences emerging between consumer and health professional/researcher groups (Figure 2). Among consumers, the greatest variability in responses was observed for notifications related to completing sleep and mood surveys (median 12, range 11‐15) and sleep goals (median 12, range 11‐15). For health professionals and researchers, the greatest variability was noted for notifications concerning step count (median 12, range 11‐14; Table 3). Notifications relating to wearing an activity tracker (median 13, range 12‐15) and logging pain scores (median 14, range 12‐15) had the highest scores for consumers, and all the other notification scores for consumers, health professionals, and researchers had a median score of 12/15. When notification scores were analyzed by individual questions (understanding, usefulness, likelihood to take action, and positive encouragement), the ratings were most similar between groups for usefulness, closely followed by encouragement. Health professionals and researchers generally provided lower ratings for ease of understanding and likelihood of taking action (Figure 2 and Table 3).

**Table 3.** Notification survey scores for each domain across 4 characteristics: understanding, usefulness, likelihood to take-action, and positive encouragement^a^.

Notification domains (n=80) and characteristics assessed	Consumers, median (IQR)	HPR^b^, median (IQR)	Combined scores, median (IQR)	Consumer total score		HPR total score		Combined total score, median (IQR)	Notifications scoring <12/15 changed (total=13), n	Notifications scoring ≥12/15	
				Median (IQR)	Min-max	Median (IQR)	Min-max			Changed (total=47), n	Unchanged (total=20), n
Step count (n=20)				12 (12-15)	6‐15	12 (11-14)	9‐15	12 (11-14)	5	9	6
Understanding^c^	5 (4-5)	4 (4-5)	5 (4-5)								
Usefulness^d^	4 (4-5)	4 (4-5)	4 (4-5)								
Likelihood to take action^e^	4 (4-5)	3 (3-4)	4 (3-4)								
Positive encouragement^f^	5 (4-5)	4 (3-5)	4 (4-5)								
Sleep goal (n=13)				12 (11-15)	8‐15	12 (11-13)	6‐15	12 (11-14)	1	11	1
Understanding	5 (4-5)	4 (4-5)	5 (4-5)								
Usefulness	4 (3-5)	4 (4-4)	4 (3-4)								
Likelihood to take action	4 (3-5)	3 (3-4)	4 (3-4)								
Positive encouragement	4 (4-5)	4 (4-5)	4 (4-5)								
Log pain scores (n=13)				14 (12-15)	3‐15	12 (11-13)	7‐15	12 (11-15)	1	7	5
Understanding	5 (4-5)	4 (4-4)	4 (4-5)								
Usefulness	4 (4-5)	4 (3-4)	4 (4-5)								
Likelihood to take action	5 (4-5)	4 (3-4)	4 (4-5)								
Positive encouragement	4 (4-5)	4 (4-5)	4 (4-5)								
Completion of surveys (sleep and mood) (n=11)				12 (11-15)	4‐15	12 (11-12)	9‐15	12 (11-14)	3	6	2
Understanding											
Sleep	5 (4-5)	4 (4-4)	4 (4-5)								
Mood	5 (4-5)	4 (4-4)	4 (4-5)								
Usefulness											
Sleep	4 (4-5)	4 (4-4)	4 (4-5)								
Mood	4 (3-5)	4 (3-4)	4 (3-5)								
Likelihood to take action											
Sleep	4 (4-5)	4 (4-4)	4 (4-5)								
Mood	4 (4-5)	4 (3-4)	4 (3-5)								
Achieving goals (n=4)				12 (12-14)	7‐15	12 (11-12)	10‐13	12 (11-13)	1	2	1
Understanding	4 (4-5)	4 (4-4)	4 (4-5)								
Usefulness	4 (4-5)	4 (4-4)	4 (4-5)								
Likelihood to take action	—^g^	—	—								
Positive encouragement	4 (4-4)	4 (3-4)	4 (4-4)								
Wearing activity tracker (n=19)				13 (12-15)	3‐15	12 (12-13)	9‐15	13 (12-15)	2	12	5
Understanding	5 (4-5)	4 (4-5)	5 (4-5)								
Usefulness	4 (4-5)	4 (4-4)	4 (4-5)								
Likelihood to take action	5 (4-5)	4 (4-4)	4 (4-5)								
Positive encouragement	4 (4-5)	4 (4-5)	4 (4-5)								

a Results are shown separately for consumers and health professionals/researchers, as well as combined group scores. Total scores (sum of 3 criteria) are also presented (median, IQR). In this table, n refers to the number of notifications (not participants).

b HPR: health professional/researcher.

c Is the information in this notification easy to understand? (“1=very difficult to understand” to “5=very easy to understand”).

d Is the information in this notification useful? (“1=not useful at all” to “5=very useful”).

e How likely are you to take-action after receiving this notification? (“1=not likely at all” to “5=very likely”).

f How would you rate the positive encouragement within this notification? (“1=very poor” to “5=excellent”).

g Not applicable.

#### Step Goal Notifications

The 20 notifications relating to step goals had a median rating of 12/15 (range 11‐14) (Table 3). Step goal notifications were rated highest for ease of understanding (median 5, range 4‐5) but had lower scores for likelihood of prompting action overall (median 4, range 3‐4), particularly from health professionals and researchers (median 3/5, range 3‐4). Qualitative feedback from consumers, health professionals, and researchers suggested that some step goal messages were clear and motivating, while others raised concerns about accessibility and tone. For instance, one consumer commented, “Clear simple statement encouraging participants to walk regularly” [Consumer]. In contrast, another noted, “I can’t walk far, and I’m pretty unsteady, so this message wouldn’t enable me to change that” [Consumer].

Colloquial language was flagged as an issue. Statements such as “you’re on fire,” which were intended to be motivating, were flagged as potentially confusing: “Some people may not understand the term ‘you’re on fire’ especially if English is not their first language or they are from an older generation” [Researcher]. Similarly, “ ‘step up the pace’ may suggest to some that they need to walk faster, is the message about accumulating more steps, or about accumulating more steps at a faster pace?” [Researcher]. As a result, this notification was revised to use simpler, more precise language.

🚶‍♂*Let’s make every step count towards a healthier you! Try adding short walks into your day to help reach your step goal.*

Consumers provided feedback that step count was not always relevant to them, *“*walking over 100 meters is painful BUT I play bowls 3 time a week at 6000 steps each game 20 meters then rest 21 times in a game” [Consumer]. Despite being active, some participants reported their activities did not translate to higher step counts: “Less steps because I’ve done an hour and half of hydrotherapy and resting today” [Consumer].

**Figure 3.**
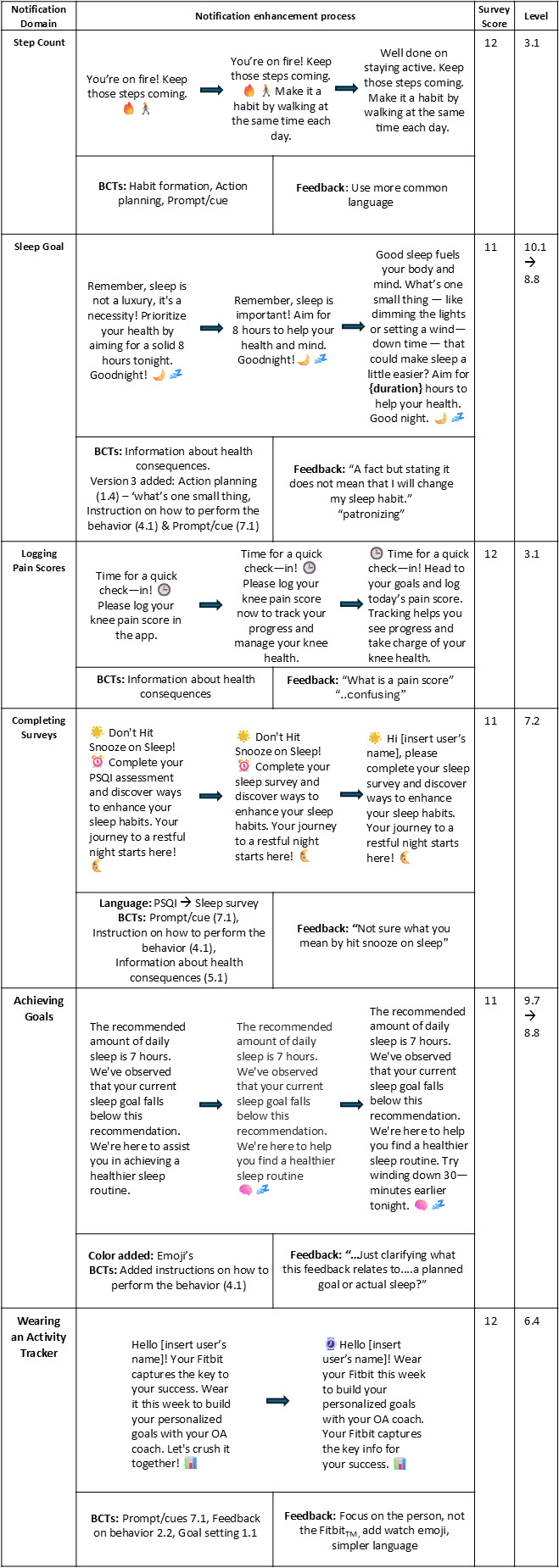
Process of notification enhancement. Examples of the notification enhancement process after consumer, health professional, and researcher feedback. BCTs=behavior change techniques (coded using the Behavior Change Technique Taxonomy v1). Feedback=examples of survey comments. Survey scores (max 15). Reading level=grade level from Sydney Health Literacy Editor (target ≤ grade 8). PSQI: Pittsburgh Sleep Quality Index.

The timing of step notifications was flagged as important: “I think the timing of this notification (step goal) would be important, not too late in the day so they still have time to take action” [Researcher]. Figure 3 shows the process of how a step goal notification was enhanced; 14 of the step goal notifications were modified after feedback.

#### Sleep Goal Notifications

The 13 sleep-related notifications had a median overall score of 12/15 (range 11‐13). Sleep goals were rated highest by consumers for ease of understanding (median 5, range 4‐5) but scored comparatively lower in perceived usefulness and likelihood to take action (median 4, range 3‐5). One participant commented, “A well-known fact, but stating it does not mean that it will make a difference to my sleep habit” [Consumer]*.* Another commented, “Sleep can be so problematic depending upon what else is happening in one’s life. I also find quality sleep difficult to measure” [Consumer].

The notifications that scored lowest for likelihood to take action by health professionals and researchers were the sleep-related notifications (median 3, range 3‐4), which had the equal lowest score with step count notifications. Qualitative feedback further contextualized these scores, noting the absence of precise guidance.

*For many people who have sleep disturbance, the “gift” of 8 hours sleep might not be possible. We could be asking them to aim for something that is out of their control. There isn’t an actionable step like: go to bed at a reasonable time or give yourself time to wind down before bed.*

[Health professional]

Out of the 13 sleep notifications, 12 were enhanced after the feedback process. Figure 3 shows an example of a sleep notification enhancement.

#### Logging Pain Score Notifications

Logging pain score notifications received a median total score of 12/15 (range 11‐15), indicating moderate acceptability overall. This was the highest consumer score for any notification type, with a median score of 14/15 (range 12‐15), whereas the health professionals and researchers assigned a lower median score of 12/15 (range 11‐13). The notification,

Have you logged your pain score today? It only takes a minute and it’s so valuable for your health journey! *📲 💪*

received a median score of 15/15, showing high acceptability. The qualitative feedback reflected these divergent views on the usefulness and psychological impact of such prompts. Some participants found these notifications helpful for fostering reflective self-management:

*I am beginning to listen to my knee pain a bit better these days...If I had a message as a reminder, I’m certain it would help or at least encourage me to stop and consider for a moment before deciding what to do.*

[Participant 16, consumer]

Others raised concerns about the potential for pain-focused prompts to reinforce negative attention to symptoms:

*Asking people to focus on the pain, of course it is painful. Some people will dwell on the pain rather than push through or raise their tolerance.*

[Participant 34, consumer]

*I would discourage rating pain levels with a chronic pain condition.*

[Participant 9, researcher]

These data suggest that while pain tracking may support adaptive pacing and awareness for some, it may negatively highlight a strong pain response in others. Of the 13 notifications logging pain scores, 8 were changed based on feedback. Figure 3 shows an example.

#### Completion of Surveys (Sleep and Mood) Notifications

A total of 11 notifications prompted users to complete a monthly sleep and mood survey. The median total score was 12/15 (range 11‐14), with similar scores for both groups. Consumers had a median score of 5/5 for perceived understanding, and all other scores were 4/5 for both groups. Among the notifications related to completing surveys, one stood out as particularly effective and required no modification throughout the iterative feedback process. The notification, “Make your screen time count, take 5 minutes to complete your sleep survey, now!” received a high overall score of 15/15, indicating high acceptability across all domains. One participant commented, “Encouraging, simple message to prompt participants to take action” [Consumer]*,* and another commented*,* “I like that the prompt indicates how long it will take” [Researcher]. This example illustrates how concise notifications that set clear expectations for time commitment can enhance engagement, even when the task is related to a potentially sensitive topic, such as sleep disturbance (Figure 3).

#### Goal Achievement Notifications

There were only 4 preset notifications to encourage users to work toward their goals. Personalized goal achievement notifications were also generated based on the activity goals specific to the OA Coach app users. Only the 4 preset notifications were evaluated and these focused on daily step count, aiming for 150 minutes of aerobic activity per week, engaging in 3 knee strengthening sessions per week, and an encouragement notification if sleep duration falls below 7 hours. The median score for these notifications was 12 (range 11‐13). Feedback from participants highlighted user assumptions about the broader app experience, with several participants noting that the effectiveness of certain notifications depended on additional in-app support. In response to the knee strengthening notification, one participant commented, “Positive clear message. One presumes there is also information provided on knee strengthening exercises” [Consumer]. Similarly, with the sleep goal notification, “I would like to think there would be some suggestions to help if this was the case?” [Consumer]*.* Out of the 4 goal achievement notifications, 3 were modified due to feedback (Figure 3).

#### Wearing the Activity Tracker

Notifications for wearing the activity tracker were more highly rated, with an overall median score of 13 (range 12‐15). Participant feedback on notifications that used device-specific terminology (Fitbit Inspire 2) highlighted that they would prefer to have the option to use other wearable technologies, “I don’t have a Fitbit so cannot comment on its usefulness” [Consumer], and “I assume that whatever app is used to read the steps taken that it will work with a range of devices, not just Fitbit. e.g., Garmin, Galaxy watch, Apple watch etc.” [Consumer]. Further comments noted the overuse of colloquial language within the notifications (eg, “crush it,” “your Fitbit is on fire,” and “keep rocking those steps”), which detracted from the simplicity of the message related to the watch. Additionally, feedback emphasized that messages should center around the user as the agent of change, rather than the technology itself.

*This message focuses on the Fitbit being the key rather than the person, but I understand that by using the Fitbit then you get data that they can then take control themselves. Not sure of the “crush it” terminology for putting a watch on.*

[Researcher]

The enhancement process for the notification mentioned is presented in Figure 3.

Feedback from the survey participants identified common reasons for suggested changes to the notifications. These included overly technical language (eg, references to “DASS-21” or “PSQI” surveys), colloquial or informal expressions (eg, “you’re on fire,” “high five,” and “you’re on a roll”), excessive or inconsistent punctuation, and unclear or confusing phrasing (eg, “let’s step up the pace...”). Some notifications were described as patronizing or frustrating, particularly those with overly enthusiastic praise (eg, “gold medal performance” and “Fantastic job...”) or unrealistic expectations (eg, “...8 hours sleep goal” and “keep those steps coming...”).

### Aim 2: The Development of Knee OA Educational Messages

#### Phase 1: Development and Enhancement of Educational Messages With BCTs and Accessible Language

The topics covered by the educational messages developed initially were as follows: understanding knee OA, the importance of staying active, healthy weight management, the benefits of strengthening exercises, exercise tips, overcoming barriers to exercise, nutrition for joint health, pain management—flare-ups, how assistive devices can help you move better, the power of good posture and staying motivated, what to know about knee surgery, and a concluding message of encouragement and goal setting.

#### Phases 2 and 3: Consumer, Health Professional, and Researcher Review, and Data Analysis and Refinement

##### Focus Groups

The consumer focus group was conducted in person with 6 participants (3 females and 3 males). The group recommended that 2 of the original 12 messages be divided into separate topics, resulting in a total of 14 educational messages. The additional topics were “Do I need a scan?” (initially titled “When should I get further investigations?”) and “Staying motivated with knee OA,” included as week 13 in the delivery sequence. A reordering of the message schedule was also advised, with the *exercise tips* message moved from week 5 to week 3, to highlight its importance in the early stages of self-management. The main topics and order of the weekly messages can be found in Multimedia Appendix 4.

Across all focus groups, a preference for a consistent message structure emerged. Participants responded positively to messages that began with a descriptive heading introducing the topic, followed by a brief introductory sentence, then 3 or 4 key points supported by relatable, practical examples, and concluded with a motivating statement with a supportive tone. This structure, reinforced with the use of bullet points and simple language, was identified as the most accessible and engaging way to present this information. Participants noted that this format helped them better understand and connect with the content. The two quotes below illustrate this:

*Set up message with opening statement then points to follow.*

[Consumer]

*Dot points are easier to follow and clearer. Sentences simplified to key points.*

[Researcher]

Participants consistently valued messages that used simple language, included relatable examples, and provided actionable advice. The following comments were made about message 2 on the importance of staying active:

*First line too vague. points also too vague, give some explanation.*

[Health professional]

*How long is too long for being inactive - can make people anxious about being still. Give some guidance if possible.*

[Health professional]

Health professionals and researchers placed stronger emphasis on ensuring the messages were evidence-based. For example, the original message “The power of good posture” (message 11) was critiqued as lacking evidence: “No evidence to support this” [Researcher] and “no research to support this” [Health professional]. One researcher suggested reframing the message to focus on mindful movement and its role during symptom flare-ups, “The power of mindful movement – reframe, could relate to flares. How does it feel? changing positions can help” [Researcher]. In response, the message was revised to “Pay attention to how you move” and included practical tips prompting users to notice how their knee feels, and to adjust movement or posture accordingly.

Several participants appreciated affirming statements that normalized setbacks within the messages, “Good to reassure about setbacks” [Researcher, message 5], and “Things can get in the way of exercise. This is normal” [Health professional, message 6]. Reinforcing that occasional lapses in health behaviors were acceptable was appreciated by consumers, “Don’t stress. Have the occasional ‘treat’ it’s o.k.!” [Consumer, message 7]. Across groups, there was a strong preference for messages that encouraged gradual behavior change, rather than setting unrealistic or rigid goals. Participants valued language that encouraged persistence, framed progress as cumulative, even if small, and built self-efficacy. Examples included “Every bit you do helps” [Consumer, message 6], “celebrate small wins” [Consumer, message 11], and “Every step is important, each step, no matter how big or small” [Researcher, message 14].

While the educational concepts proposed by French et al [53] provided a strong foundation for developing messages, several terms, such as “overweight” and “damage,” generated discussion during the focus groups. These terms, though clinically conventional, were highlighted as unhelpful. Health professionals and researchers preferred “weight management” versus “overweight” to encompass individuals across the weight spectrum, including those of normal or underweight status. Health professionals also recommended changing “joint damage on an x-ray” to a more neutral tone “joint changes on an x-ray.” Consumers did not specifically comment on the term “damage”; they suggested that the draft message, which covered both investigations and assistive devices (eg, walking sticks, knee braces, and shoe inserts), be separated for simplicity. In response, a new message titled “How assistive devices can help you move” was developed.

Message 12, which focused on “what to know about knee surgery,” warranted particular attention. Consumers emphasized the importance of clearly communicating that surgery does not always help, that rehabilitation can be challenging, and that better outcomes are associated with being physically stronger beforehand.

*Rehab is not for the faint-hearted. You’re not escaping exercise by having surgery.*

[Consumer]

*Surgery doesn’t always help.*

[Consumer]

*Exercise is important at all stages of OA, and the stronger you are going into surgery, the better the outcome.*

[Consumer]

Health professionals and researchers agreed with these key points; they further highlighted the need to encourage shared decision-making and suggested the inclusion of example questions patients could ask their health care team. They strongly recommended that the message include a clear statement reflecting international guidelines, which advised against the use of arthroscopy for knee OA due to its lack of effectiveness.

*The decision to have surgery is a conversation to have with your health professional team give examples of questions.*

[Researcher]

*Message re arthroscopy very clear - recommended against. Not effective for knee OA.*

[Researcher]

*A viable treatment option for the right person needs to be discussed with your healthcare team. Even if you have it, it doesn’t always help.*

[Health professional]

##### Educational Message Final Survey

**Figure 4.**
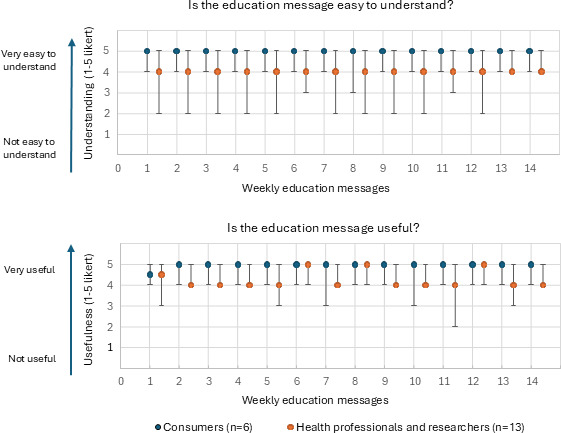
Median (minimum and maximum) ratings for each education message by group: perceived ease of understanding and usefulness.

Participants in the focus groups provided feedback on the revised educational messages in a survey. Figure 4 shows that median score ratings for consumers were consistently high; they rated all messages more favorably than health professionals, with a median score of 5/5 across all messages for both understanding and usefulness. In contrast, the health professionals and researchers provided variable scores, particularly for perceived usefulness. Although their median ratings tended to cluster around 4 out of 5. Several messages received lower scores and showed greater variability, as indicated by the wider range of responses (eg, messages 6, 8, 12, and 13), and these were revised further based on this feedback.

## Discussion

### Principal Findings

This study outlines the theory-informed iterative co-design process used to enhance notifications and develop educational messages intended to prompt users of the OA Coach app to adopt and sustain healthier behaviors for self-management of their knee OA. We grounded the design of the notifications and educational messages in behavior change theory, specifically the Behavior Change Technique Taxonomy [46] and COM-B model [55,63], to ensure that each notification and educational message addressed the key drivers of behavior. We sought consumer and expert feedback in the development of the OA Coach app notifications and educational messages using surveys and focus groups to improve their acceptability, clarity, usefulness, and tone. These qualities have been associated with improved user motivation, satisfaction, and engagement in prior research with digital health interventions [64-66].

There were similarities and differences in our findings from the surveys and focus groups with consumers compared with health professionals and researchers. A common finding was that colloquial language, used initially to add variety and positivity to the notifications, was often perceived to be confusing and distracted from the main message. In contrast, there was some variation in responses to the survey questions on mood and sleep. While median ratings of acceptability were similar between the consumer and the health professional/researcher groups, the consumer scores were more widely distributed, indicating that some consumers found these notifications more useful than others. There were greater differences between responses of the consumers and health professional/researcher groups related to the JITAI-informed daily pain score logging component of the OA Coach. While the pain logging notifications were rated favorably by consumers, the health professionals and researchers expressed concerns that daily logging might inadvertently reinforce hypervigilance or anxiety in some individuals with persistent OA pain [67]. These concerns are consistent with the broader pain literature, highlighting that continual self-monitoring of symptoms can sometimes exacerbate attention to pain [67]. Our findings underscore the importance of designing tracking features and associated notifications that balance the theoretical and practical benefits of adaptive, personalized feedback with user burden and perceived relevance [39,40]. Ensuring that the data collected contributes meaningfully to the user’s personal goals and immediate self-management needs is likely to enhance engagement with, and the effectiveness of, digital behavior change interventions like OA coach [40].

The longer weekly OA Coach App educational messages were developed to provide more in-depth information, practical strategies, and motivational support, reinforcing self-management behaviors. One output of the co-design focus groups was the emergence of a preferred layout, using short sentences in the active voice, at grade 8 reading level, with a descriptive heading, brief introduction, key points with practical examples, and an encouraging closing statement. We anticipate that the collaborative approach used to develop these messages will enhance comprehension, engagement, and empowerment of OA Coach app users.

Health professionals highlighted the importance of language choice, particularly with terms such as “joint damage” and “overweight,” preferring “joint changes” and “weight management.” Emerging literature suggests that biomedical language, such as “damage” and “wear and tear,” can reinforce unhelpful beliefs about structural deterioration, potentially increasing the fear of movement and reducing engagement in self-management strategies [20,68-73]. In their systematic review, Behera et al [74] emphasized that OA education is a complex relational process influenced by multidimensional factors and that empowering language can enhance patient engagement and agency. These insights align with the objectives of the Changing the Narrative on Osteoarthritis initiative, which calls for a shift from negative, condition-focused discourse toward language that supports resilience, autonomy, and self-efficacy in people living with OA [73]. In response to feedback from focus groups, surveys, and the mentioned research, educational messages were revised to reflect more empowering, hopeful language, while preserving the original evidence-based content [7,9,53,75].

### Strengths and Limitations

The inclusion of consumers, health professionals, and researchers offered considerable strengths to the development process. Consumers represented a broad age range (46‐85 years) and brought lived experience of knee OA, while the inclusion of health professionals and researchers added multidisciplinary perspectives and expert insight. High levels of educational attainment across all groups contributed to rich, thoughtful feedback, particularly regarding message clarity, tone, and relevance. However, the sample lacked cultural and linguistic diversity, with no representation from Aboriginal and Torres Strait Islander peoples, and limited representation of those from Pacific, African, or Latin American backgrounds. The higher education levels observed across the sample likely meant that participants were more comfortable with complex written material, although they still highlighted the need for grade 8 reading levels. This is supported by research demonstrating a strong association between higher education attainment and greater health literacy [76]. As such, our findings related to message readability may not be generalized to populations with lower education levels or limited health or digital literacy, who often experience greater difficulty understanding and accessing health information.

The small sample sizes for each group (consumers, health professionals, and researchers) and the exploratory nature of this study also limit its generalizability as the feedback may not have captured the full diversity of perspectives across age, digital literacy, health literacy, or OA severity. Although consumer feedback was prioritized throughout educational message development, and messages were adapted accordingly before being reviewed by health professionals and researchers, final decisions about edits were made by the research team. This may have inadvertently favored certain stakeholder views over others.

Another limitation was that some of the participants did not experience using the OA Coach app prior to providing feedback. Instead, they reviewed notifications and messages through printed handouts, digital documents, and focus group discussions. It is possible that engaging with the app in real-world conditions may have offered additional insights, as actual use involves contextual factors such as screen size, multitasking (interacting with the app while attending to other tasks or receiving alerts from multiple apps simultaneously), and notification timing. Therefore, while the focus groups and surveys allowed for rich discussion and language refinement, the extent to which these findings translate into behavior change in real-world settings remains uncertain. Nevertheless, this study provides a clear, reproducible method for patient-centered co-design of notifications and messages in mHealth interventions. This approach supports clarity, acceptability, and relevance for people using an mHealth app. Researchers and app developers can use these practical insights in their work as guidance for designing and refining patient-focused messages in mHealth contexts (Textbox 1).

Textbox 1. **Practical tips for writing educational messages in mobile health.**

✓ Descriptive subject heading

✓ Short introductory sentence

✓ 3-4 key bullet points

✓ Relatable, everyday examples

✓ Encouraging closing statement

✓ Active voice

✓ Grade 8 readability

✕ Avoid colloquial/slang expressions

✕ Avoid medical terms

### Considerations for Future Research

Future research will evaluate these notifications and educational messages within the live OA Coach app to assess how users interact with content in real time. In addition, examining how different subgroups respond to variations in tone, format, and timings of notifications and messages will help inform more personalized digital health communication strategies, including the needs of culturally and linguistically diverse populations, to ensure these strategies are inclusive and responsive to diverse communication needs.

Currently, the OA Coach app offers semitailored support; however, advances in artificial intelligence will enable greater individualization. For example, by collecting additional information at the start of app use, such as current user activity levels, coexisting health conditions, user goals, and the topics a person most wants to learn about, the program could adapt both the type and order of messages to better align with individual needs. Future research could examine whether this type of approach enhances user engagement and self-management outcomes, while also considering important factors such as privacy and user trust. The updated OA Coach app is currently being evaluated in the COASTAL [77] randomized controlled trial, which compares face-to-face, telehealth, and digitally delivered models of care for knee OA.

### Conclusions

This study developed and refined evidence-based notifications and educational messages for knee OA that were grounded in behavior change theory and aligned with international guideline recommendations. Through an iterative co-design process involving consumers, health professionals, and researchers, we identified key preferences for message content, tone, format, and structure. These insights informed the development of 14 educational messages and the refinement of 80 notifications for integration into the OA Coach app.

## Supplementary material

10.2196/83507 Multimedia Appendix 1 Behavior change techniques applied in notifications for the OA Coach app.

10.2196/83507 Multimedia Appendix 2 Radar charts of notification scores by domain and evaluation criteria comparing the consumer group with the health professional and researcher group. Six radar charts showing median notification scores across 6 domains: step count, sleep goals, logging pain scores, goal achievement, completion of surveys (sleep and mood), and wearing an activity tracker. Step count, sleep goals, logging pain scores, and tracker domains were rated on 4 criteria (understanding, usefulness, encouragement, and ability to prompt action). Goal achievement and survey completion were rated on 3 of the criteria. Results are shown for consumers (blue) and health professionals/researchers (orange). Scores range from 0 (center) to 5 (outer edge), with higher values indicating more positive evaluations. For goal achievement notifications, both groups gave identical ratings.

10.2196/83507 Multimedia Appendix 3 Focus group plan and open-ended questions.

10.2196/83507 Multimedia Appendix 4 Education message topics for the OA Coach app.

10.2196/83507 Checklist 1 STROBE checklist.

## Abbreviations

BCT: behavior change technique
COM-B: capability, opportunity, motivation—behavior model
DASS-21: 21-Item Depression, Anxiety, and Stress Scale
JITAI: just-in-time adaptive intervention
mHealth: mobile health
OA: osteoarthritis
OACRG: Osteoarthritis Clinical Research Group
PSQI: Pittsburgh Sleep Quality Index
REDCap: Research Electronic Data Capture
SHeLL: Sydney Health Literacy Lab Editor
STROBE: Strengthening the Reporting of Observational Studies in Epidemiology
