# Supplementary material for: Messages and Notifications for the “OA Coach” Knee Osteoarthritis Self-Management Mobile App: Codevelopment and Evaluation Using a Participatory Research Design With Focus Groups and Surveys
Source: J Med Internet Res. 2026 May 4;28:e83507. doi: 10.2196/83507 (PMC13138410; doi:10.2196/83507)
Supplement: Multimedia Appendix 1 [file jmir-v28-e83507-s001.docx]

| **Behavior Change Techniques Applied in Notifications (Based on BCT taxonomy v1)** *(1)* | |
| --- | --- |
| Goals and planning | |
| 1.1 | Goal setting (behavior) |
| 1.2 | Problem solving |
| 1.4 | Action planning |
| 1.6 | Discrepancy between current behavior and goal |
| Feedback and monitoring | |
| 2.2 | Feedback on behavior |
| 2.3 | Self-monitoring of behavior |
| 2.4 | Self-monitoring of outcomes of behavior |
| 2.6 | Biofeedback |
| Social support | |
| 3.1 | Social support (unspecified) |
| Shaping knowledge | |
| 4.1 | Instructions on how to perform a behaviour |
| Natural consequences | |
| 5.1 | Information about health consequences |
| 5.6 | Information about emotional consequences |
| Associations | |
| 7.1 | Prompts/cues |
| Repetition and substitution | |
| 8.2 | Behavior substitution |
| 8.3 | Habit formation |
| 8.7 | Graded tasks |
| Comparison of outcomes | |
| 9.1 | Credible source |
| 9.2 | Pros and cons |
| Rewards and threats | |
| 10.4 | Social reward (positive reinforcement) |
| 10.9 | Self-reward |
| Self-belief | |
| 15.1 | Verbal persuasion about capability |
| 15.3 | Focus on past success |
| 15.4 | Self-talk |

1. Michie S, Richardson M, Johnston M, Abraham C, Francis J, Hardeman W, et al. The behavior change technique taxonomy (v1) of 93 hierarchically clustered techniques: building an international consensus for the reporting of behavior change interventions. Ann Behav Med. 2013;46(1):81-95.
