# Supplementary figures and images for: Messages and Notifications for the “OA Coach” Knee Osteoarthritis Self-Management Mobile App: Codevelopment and Evaluation Using a Participatory Research Design With Focus Groups and Surveys
Source: J Med Internet Res. 2026 May 4;28:e83507. doi: 10.2196/83507 (PMC13138410; doi:10.2196/83507)

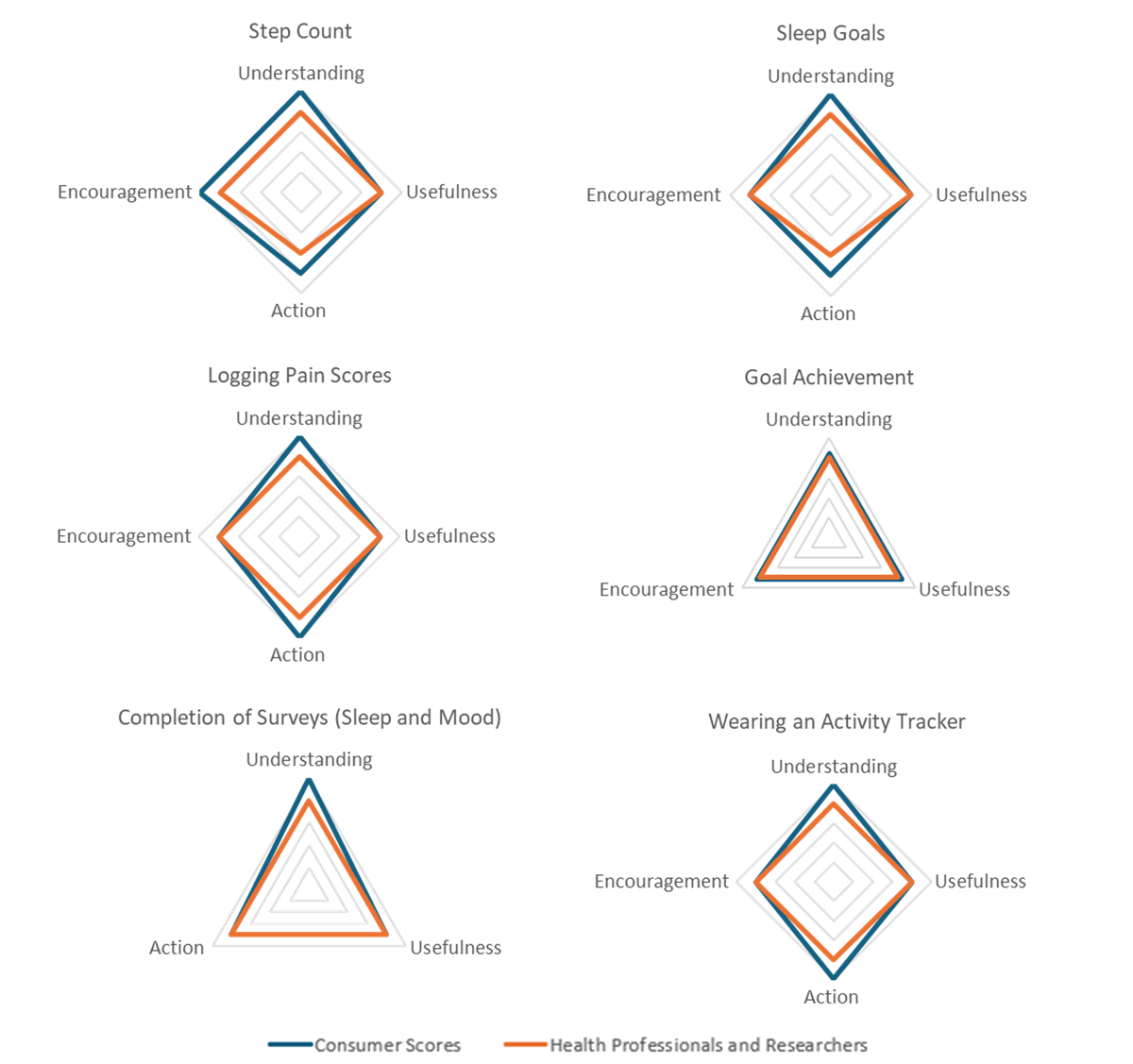

Supplement: Multimedia Appendix 2 [file jmir-v28-e83507-s002.png]
