# Supplementary material for: Messages and Notifications for the “OA Coach” Knee Osteoarthritis Self-Management Mobile App: Codevelopment and Evaluation Using a Participatory Research Design With Focus Groups and Surveys
Source: J Med Internet Res. 2026 May 4;28:e83507. doi: 10.2196/83507 (PMC13138410; doi:10.2196/83507)
Supplement: Multimedia Appendix 3 [file jmir-v28-e83507-s003.docx]

# Focus Group Plan

### Objective

1. To develop a bank of messages providing advice, information, and motivation to encourage long-term positive lifestyle choices.
   1. Ensure alignment with international guidelines.
   2. Incorporate behavior change techniques and positive language
   3. Include practical tips for making lifestyle changes
   4. Determine the optimal order and timing of messages for the programme

### Introduction (10-15 minutes)

- Welcome and Introductions: Introduce researchers and participants.
- Purpose of the session: Explain the goals and importance of the focus group.
- Ground rules: Establish guidelines, aiming for 10 minutes per message, encouraging participation from all attendees.
- Consent: Confirm that participants understand their rights and have provided informed consent.

### Discussion of educational content of messages (2 hours)

- Presentation of messages: Present each educational message, focusing on content.
- Guided Discussion: to explore participants' views on the content, clarity, and relevance of each message.
- Sample Questions:
  - How clear and understandable was the message?
  - What parts of the message stood out as particularly relevant or helpful?
  - Is there anything missing that you think should be included?
  - What are your overall thoughts on the messages as a whole?

### Behavior Change Techniques and Practical Tips

- **Behavior Change Focus:** Identify and evaluate the behavior change techniques used in the messages.
- **Practical tips:** Gather feedback on the practical tips provided and suggestions for additional tips or improvements.
- **Effectiveness, applicability and Practicality**: Discuss how practical and applicable these techniques are for promoting long-term behavior change.
- Sample Questions:
  - What behavior change techniques do you recognize in these messages?
  - How practical are these techniques in everyday life?
  - What other techniques could enhance these messages?

### Break (15-20 minutes)

- There will be a break scheduled halfway through the session with light refreshments.

### Ordering and Sequencing Discussion (20 minutes)

### Programme Context: Discuss creating a logical flow for a 12-week programme.

- Order of messaging Review: Collaboration on the sequence of the messages.
- Timing of messaging review: Collaboration on the timing of the messages.
- Guided Questions:
  - Considering the content we’ve discussed, which messages should be delivered first to set the stage?
  - How can the messages be arranged to build understanding and engagement?
  - Which messages are best suited for later stages to reinforce learning and motivate continued participation?
  - What are your thoughts on spacing messages out over 6 months (current plan is 1wk for the first 12 weeks?
  - Another option would be to start with more frequent messages in the first month to build a foundation of knowledge and then spacing them out over time. How does this approach sound to you?
  - What day and time of day should we send the weekly education message?
  - Does this plan sound like a good balance between providing enough information without overwhelming users over the 6 months?

### Wrap up and thank you (10 minutes)

- Summary: Recap the main points discussed and thank participants for their time and input
- Next steps: Inform participants that the education messages will be modified according to their feedback and added to the notification survey for them to review for final feedback.
- Finish with a post-session survey to gather additional anonymous feedback on the focus group process itself, to help with refining future sessions.
