# Supplementary material for: Messages and Notifications for the “OA Coach” Knee Osteoarthritis Self-Management Mobile App: Codevelopment and Evaluation Using a Participatory Research Design With Focus Groups and Surveys
Source: J Med Internet Res. 2026 May 4;28:e83507. doi: 10.2196/83507 (PMC13138410; doi:10.2196/83507)
Supplement: Multimedia Appendix 4 [file jmir-v28-e83507-s004.docx]

| Education Message Topics and Weekly Order  *These messages are sent out once a week to the inbox of the OA Coach Mobile app. The sending of messages is activated when the user of the app completes the baseline measurement week.* (1) | |
| --- | --- |
| **Week​** | **Education Message Topics​** |
| **1​** | Understanding knee OA​ |
| **2​** | The importance of staying active​ |
| **3​** | Exercise tips 'sore but safe'​ |
| **4​** | Healthy weight, healthy knees​ |
| **5​** | The benefits of strengthening exercises​ |
| **6​** | Overcoming challenges to exercise​ |
| **7​** | Nutrition for joint health​ |
| **8​** | How to manage sudden changes in OA symptoms​ |
| **9​** | How assistive devices can help you move​ |
| **10​** | Do I need a scan?​ |
| **11​** | Pay attention to how you move​ |
| **12​** | What to know about knee surgery​ |
| **13​** | Staying motivated with knee OA​ |
| **14​** | Congratulations on 14-weeks of knee care​ |

1. Bryce KL, Eyles JP, Bowden JL, Hunter DJ, Bloul N, Huang R, Duong V. DEVELOPMENT OF A BANK OF THEORY-INFORMED AUTOMATED NOTIFICATIONS FOR THE OA COACH MOBILE APPLICATION DESIGNED TO SUPPORT BEHAVIOUR CHANGE IN THE SELF-MANAGEMENT OF KNEE OSTEOARTHRITIS. Osteoarthritis and Cartilage. 2025;33:S180.
