# Supplementary material for: Messages and Notifications for the “OA Coach” Knee Osteoarthritis Self-Management Mobile App: Codevelopment and Evaluation Using a Participatory Research Design With Focus Groups and Surveys
Source: J Med Internet Res. 2026 May 4;28:e83507. doi: 10.2196/83507 (PMC13138410; doi:10.2196/83507)
Supplement: Checklist 1 [file jmir-v28-e83507-s005.docx]

STROBE Statement—checklist of items that should be included in reports of observational studies

|  | Item No. | Recommendation | Page  No. | | Relevant text from manuscript |
| --- | --- | --- | --- | --- | --- |
| **Title and abstract** | 1 | (*a*) Indicate the study’s design with a commonly used term in the title or the abstract | 1 | | Mixed-methods participatory design research using focus groups and online surveys. |
|  |  | (*b*) Provide in the abstract an informative and balanced summary of what was done and what was found | 2 | | Methods: This mixed-methods study used a three phase process for improving existing notifications and developing new educational messages: (1) initial development and/or enhancement using behavior change techniques and accessible language, (2) expert review, and (3) refinement based on participant feedback.  We enhanced the existing notifications to incorporate behavior change theory and improve readability, then we assessed ease of understanding, usefulness and motivational impact in an online survey using 1-5 Likert scales and free text responses. Median scores and interquartile ranges were calculated, free text responses were summarized using content analysis, and notifications that scored ≥12/15 and/or had two or more consistent recommendations in free text responses were systematically refined.  The new educational messages were initially drafted based on OA clinical practice guidelines, then reviewed in three focus groups (in person and online) for detailed guidance on topic selection, language and timing. Content analysis of focus group data informed systematic refinement of the messages. The refined messages were assessed for understanding and usefulness in an online survey (1-5 Likert scales) and free text responses, and refined further based on survey results.  Results: Fifty-seven participants assessed the existing notifications in the survey. Consumers rated notifications relating to sleep and mood the lowest, and those encouraging logging pain scores the highest. Health professionals and researchers reported a median score of 12/15 across all notifications, with greatest variability observed for step count, activity tracker use and pain score logging. Eighty notifications were refined to enhance clarity, engagement, and effectiveness in prompting meaningful actions by future OA Coach users.  Six consumers, seven health professionals and six researchers participated in three focus groups to co-design fourteen educational messages. A preference for a consistent message structure emerged: a descriptive heading, a brief introductory sentence, followed by three or four key points with practical examples concluded with a motivating statement.  Key themes guiding modifications for both notifications and educational messages included reducing technical jargon, simplifying colloquial expressions, and removing language perceived to be patronizing or frustrating. |
| Introduction | | | |  | |
| Background/rationale | 2 | Explain the scientific background and rationale for the investigation being reported | 2-3 | | Scalable solutions are needed to address these challenges, that improve public access to guideline-recommended care. Digital health technologies, such as mobile health (mHealth) apps, offer a promising approach to overcome these barriers by providing accessible, low-cost, and evidence-based interventions that can be delivered at scale. Digital technology using mobile phones is a fast-growing field that promotes adherence to treatment in self-managing chronic conditions, including OA [32-35]. The World Health Organization (WHO) identified digital health as a global priority, emphasizing that digital health can support equitable and universal access to quality health services and improve health outcomes [4, 36]. Implementing digital health technologies, such as the OA Coach has significant potential to improve access to the best evidence care for people living with knee OA. |
| Objectives | 3 | State specific objectives, including any prespecified hypotheses | 3 | | The two specific aims were:  ​1.  Enhance the original OA Coach app call-to-action and encouragement notifications by applying behavior change theory, refining, revieing and adapting them with consumers, health professionals and researchers.  ​2. Co-develop new knee osteoarthritis educational messages based on international osteoarthritis guidelines using focus groups and surveys with consumers, health professionals and researchers. |
| Methods | | | |  | |
| Study design | 4 | Present key elements of study design early in the paper | 4 | | The mixed-methods study design was based on two previously published frameworks for digital intervention development [28, 29] and adapted to include three phases: (1) initial development and/or enhancement with BCTs and accessible language, (2) expert review, and (3) refinement based on participant feedback |
| Setting | 5 | Describe the setting, locations, and relevant dates, including periods of recruitment, exposure, follow-up, and data collection | 4  8 | | The study was conducted at the Kolling Institute, the University of Sydney (Dec 2024-Mar 2025)  The first focus group for consumers was three hours in duration, held in-person at the Kolling Institute, University of Sydney. The second and third focus groups were 2 hours in duration, held online with health professionals and researchers, respectively. |
| Participants | 6 | (*a*) *Cohort study*—Give the eligibility criteria, and the sources and methods of selection of participants. Describe methods of follow-up  *Case-control study*—Give the eligibility criteria, and the sources and methods of case ascertainment and control selection. Give the rationale for the choice of cases and controls  *Cross-sectional study*—Give the eligibility criteria, and the sources and methods of selection of participants | 4-5 | | People were eligible if they had been diagnosed with knee osteoarthritis (OA), aged 18 years or older, spoke fluent English and owned a smartphone. Previous participants from the feasibility study were contacted to be part of this study. Consumers may or may not have used the OA Coach app previously during the 6-week feasibility study [27]. Health professionals and researchers were eligible if they had experience working with people with knee OA. Specifically, health professionals were eligible if they treated people with OA and researchers were expected to have published at least two research papers in OA management. All participants provided informed consent. Demographic data collected included age, gender, ancestry, education level and years of work experience (health professionals and researchers).  Recruitment was conducted through the Osteoarthritis Clinical Research Group (OACRG) volunteer database. Health professionals and researchers were also approached via email using the researchers’ (JE and JB) clinical networks.  Purposeful sampling was employed |
|  |  | (*b*) *Cohort study*—For matched studies, give matching criteria and number of exposed and unexposed  *Case-control study*—For matched studies, give matching criteria and the number of controls per case |  | | N/A |
| Variables | 7 | Clearly define all outcomes, exposures, predictors, potential confounders, and effect modifiers. Give diagnostic criteria, if applicable | 4-9 | | Outcomes were surveys and focus groups and exposures were notifications and messages  N/A: predictors, potential confounders, effect modifiers and diagnostic criteria |
| Data sources/ measurement | 8* | For each variable of interest, give sources of data and details of methods of assessment (measurement). Describe comparability of assessment methods if there is more than one group | *4-9* | | Surveys and Focus Groups |
| Bias | 9 | Describe any efforts to address potential sources of bias | 4-5  7  23 | | Selection bias was minimized by using a pre-defined eligibility criteria and recruiting participants through multiple channels to increase reach  There were 80 notifications to get a rating. To minimize participant burden these were separated into 8 surveys. These Surveys were randomly allocated to participants using the REDCap data collection system.  Surveys were also completed anonymously and the data de-identified to prevent feedback being linked to specific individuals, reducing researcher bias  “final decisions about edits were made by the research team. This may have inadvertently favored certain stakeholder views over others.” |
| Study size | 10 | Explain how the study size was arrived at | 7 | | The sample size was chosen to ensure that each of the 80 notifications (distributed across eight discrete surveys) would be reviewed by a minimum of five consumers and two health professionals or researchers. |

Continued on next page

| Quantitative variables | 11 | Explain how quantitative variables were handled in the analyses. If applicable, describe which groupings were chosen and why | 7  12 (Table 2) | Descriptive statistics (median and interquartile range) were calculated for three of the four characteristics (understanding, usefulness, likelihood of taking action, and positive encouragement) within each notification domain (Table 2)  Given the small sample size (n = 17), we used median values to represent central tendency and reported the minimum and maximum scores to describe the full range of responses. Results were analyzed separately for consumers and for health professionals/researchers, then combined. All quantitative data were analyzed using Microsoft Excel. |
| --- | --- | --- | --- | --- |
| Statistical methods | 12 | (*a*) Describe all statistical methods, including those used to control for confounding |  | N/A |
|  |  | (*b*) Describe any methods used to examine subgroups and interactions | 7  13 (Table 2) | Results were analyzed separately for consumers and for health professionals/researchers, then combined.  As above |
|  |  | (*c*) Explain how missing data were addressed |  | All focus groups completed the final education message survey and only 2 data points missing from the notification surveys (<5%), so nothing was done to address missing data |
|  |  | (*d*) *Cohort study*—If applicable, explain how loss to follow-up was addressed  *Case-control study*—If applicable, explain how matching of cases and controls was addressed  *Cross-sectional study*—If applicable, describe analytical methods taking account of sampling strategy |  |  |
|  |  | (*e*) Describe any sensitivity analyses |  | N/A |
| Results | | | | |
| Participants | 13* | (a) Report numbers of individuals at each stage of study—eg numbers potentially eligible, examined for eligibility, confirmed eligible, included in the study, completing follow-up, and analysed | 9-10 | Table 1 and Figure 2  Sixty study participants took part, comprising of 40 consumers, 10 health professionals and 10 researchers. |
|  |  | (b) Give reasons for non-participation at each stage |  | None |
|  |  | (c) Consider use of a flow diagram | 10 | Figure 2 |
| Descriptive data | 14* | (a) Give characteristics of study participants (eg demographic, clinical, social) and information on exposures and potential confounders | 11 | Table 1 |
|  |  | (b) Indicate number of participants with missing data for each variable of interest |  | None |
|  |  | (c) *Cohort study*—Summarize follow-up time (eg, average and total amount) |  | N/A |
| Outcome data | 15* | *Cohort study*—Report numbers of outcome events or summary measures over time |  | *N/A* |
|  |  | *Case-control study—*Report numbers in each exposure category, or summary measures of exposure |  | *N/A* |
|  |  | *Cross-sectional study—*Report numbers of outcome events or summary measures | *10-11*  *Figure 2 and Table 1* | *60 people completed the notification surveys and 19 people took part in the focus groups and education message survey. There were 8 notification surveys with 4-5 people completing each survey and 1 education message survey completed by 19 focus group participants..s* |
| Main results | 16 | (*a*) Give unadjusted estimates and, if applicable, confounder-adjusted estimates and their precision (eg, 95% confidence interval). Make clear which confounders were adjusted for and why they were included | 11 | A total of 80 notifications were assessed in the surveys. Of these, 16% (n = 13) scored below the 12/15 threshold and were revised based on participant feedback, while 25% (n = 20) were retained unchanged, having scored 12/15 or higher and received positive feedback. An additional 59% (n = 47) of notifications with scores 12/15 or higher were also refined in response to open-text feedback, which highlighted areas for improvement in clarity, tone, and grammatical accuracy. |
|  |  | (*b*) Report category boundaries when continuous variables were categorized |  | N/A |
|  |  | (*c*) If relevant, consider translating estimates of relative risk into absolute risk for a meaningful time period |  | N/A |

| Other analyses | 17 | Report other analyses done—eg analyses of subgroups and interactions, and sensitivity analyses | |  |  | N/A |
| --- | --- | --- | --- | --- | --- | --- |
|  | | Discussion | | | | |
| Key results | 18 | Summarize key results with reference to study objectives | | 21 |  | This study outlines the theory informed iterative co-design process used to enhance notifications and develop educational messages intended to prompt users of the OA Coach app to adopt and sustain healthier behaviors for self-management of their knee OA. We grounded the design of the notifications and educational messages in behavior change theory, specifically the BCT Taxonomy [43] and COM-B model [52, 60], to ensure that each notification and educational message addressed the key drivers of behavior. We sought consumer and expert feedback in the development of the OA Coach app notifications and educational messages using surveys and focus groups to improve their acceptability, clarity, usefulness, and tone. |
| Limitations | 19 | Discuss limitations of the study, taking into account sources of potential bias or imprecision. Discuss both direction and magnitude of any potential bias | | 22-23 |  | A limitation is that the sample lacked cultural and linguistic diversity, with no representation from priority Australian populations, including Aboriginal and Torres Strait Islander peoples, and limited representation of those from Pacific, African or Latin American backgrounds. Additionally, the high education levels observed across the sample likely meant that participants were more comfortable with complex written material. This is supported by research demonstrating a strong association between higher education attainment and greater health literacy [72]. As such, findings related to message readability may not be generalizable to populations with lower education levels or limited health literacy, who often experience greater difficulty understanding health information.  Section: Limitations |
| Interpretation | 20 | Give a cautious overall interpretation of results considering objectives, limitations, multiplicity of analyses, results from similar studies, and other relevant evidence | | 24 |  | Conclusion. This study developed and refined evidence-based notifications and educational messages for knee OA, that were grounded in behavior change theory and aligned with international guideline recommendations. Through an iterative co-design process involving consumers, health professionals, and researchers, we identified key preferences for message content, tone, format, and structure. These insights informed the development of 14 educational messages and the refinement of 80 notifications for integration into the OA Coach app. |
| Generalisability | 21 | Discuss the generalisability (external validity) of the study results | | 22 |  | This study provides a clear, reproducible method for patient-centred co-design of notifications and messages in mHealth interventions. This approach supports clarity, acceptability, and relevance for people using an mHealth app. Researchers and app developers can use these practical insights in their work as guidance for designing and refining patient-focused messages in mHealth contexts (Table 3). |
| Other information | |  |  | | | |
| Funding | 22 | Give the source of funding and the role of the funders for the present study and, if applicable, for the original study on which the present article is based | | 24 |  | The OA Coach app has been funded with the help of the Lenity Foundation. Kate Bryce is supported by the Postgraduate Research in Osteoarthritis Research Scholarship, University of Sydney. |

*Give information separately for cases and controls in case-control studies and, if applicable, for exposed and unexposed groups in cohort and cross-sectional studies.

**Note:** An Explanation and Elaboration article discusses each checklist item and gives methodological background and published examples of transparent reporting. The STROBE checklist is best used in conjunction with this article (freely available on the Web sites of PLoS Medicine at http://www.plosmedicine.org/, Annals of Internal Medicine at http://www.annals.org/, and Epidemiology at http://www.epidem.com/). Information on the STROBE Initiative is available at www.strobe-statement.org.
